# Nuclear pore complexes as hubs for gene regulation

**DOI:** 10.1080/19491034.2017.1395542

**Published:** 2018-01-30

**Authors:** Maximiliano A. D'Angelo

**Affiliations:** Sanford Burnham Prebys Medical Discovery Institute, Development, Aging and Regeneration Program, NCI-Designated Cancer Center, 10901 N. Torrey Pines Road, La Jolla, CA, United States

**Keywords:** Nuclear pore complex, Nucleoporin, nuclear transport, nuclear envelope, gene expression, transcription, Muscle

## Abstract

Nuclear pore complexes (NPCs), the channels connecting the nucleus with the cytoplasm, are the largest protein structures of the nuclear envelope. In addition to their role in regulating nucleocytoplasmic transport, increasing evidence shows that these multiprotein structures play central roles in the regulation of gene activity. In light of recent discoveries, NPCs are emerging as scaffolds that mediate the regulation of specific gene sets at the nuclear periphery. The function of NPCs as genome organizers and hubs for transcriptional regulation provides additional evidence that the compartmentalization of genes and transcriptional regulators within the nuclear space is an important mechanism of gene expression regulation.

## Introduction

The genome of eukaryotes is contained inside the largest cellular organelle, the nucleus. Within the nucleus, the chromosomes are not randomly distributed but occupy defined chromosome territories.^1^ The relative position of these territories varies among different cell types and has been shown to change depending on the differentiation and metabolic state of the cell.^2^ A large amount of evidence indicates that the position of genes in the nuclear space can significantly affect their activity, and the general consensus is that gene-rich chromosomes are more centrally located than gene-poor chromosomes, which localize more towards the nuclear periphery. How nuclear organization is achieved and faithfully maintained in cells is not entirely clear, but substantial evidence has shown that nuclear envelope (NE) structures, including the nuclear lamina and the nuclear pore complexes, play an important role in this process. While it is clear that the nuclear lamina mostly associates with silent chromatin and plays a key role in gene repression,^3^ NPCs have been found to bind silent as well as active chromatin domains, and to play a negative and positive role in transcriptional regulation.^4^ These findings speak of the versatility of NPCs in regulating genome organization and function.

## Nuclear envelope and nuclear pore complexes

The nuclear envelope is a double membrane structure that surrounds the genome separating it from the cytoplasm. It is composed of an outer nuclear membrane (ONM), which is contiguous with the rough endoplasmic reticulum, and an inner nuclear membrane (INM), which is characterized by a distinct set of proteins that specifically localize to this domain, known as nuclear envelope transmembrane proteins or NETs.^5^ Underneath the INM is a meshwork of intermediate filaments known as the nuclear lamina.^3^ Nuclear pore complexes or NPCs, are large protein channels that penetrate the nuclear envelope connecting the nucleus to the cytoplasm.^6^ Being the sole gateway into the nuclear space, NPCs facilitate the exchange of most molecules between the nucleus and the cytoplasm. But in addition to their traditional function as regulators of nucleocytoplasmic molecule exchange, NPCs have recently emerged as important players in genome organization, the maintenance of genome integrity, and the modulation of gene expression.^7,8^

NPCs are built by the repetition of 32 different proteins known as nucleoporins.^9^ Traditionally, NPCs were considered structures of ubiquitous composition, but our Lab and others have recently shown that the expression of several nucleoporins varies among different cell types and tissues,^10–14^ and that changes in the composition of these structures are employed to regulate cell differentiation and tissue physiology.^11,15^ These exciting findings not only expose the existence of tissue-specific NPCs but also indicate that these channels are dynamic and modular structures that can be modified to change their properties and functions.

## NPCs in transcriptional regulation

Because NPCs are the only gateway into the nucleus, for a long time after their discovery most studies were focused on understanding the structural and physical properties of these channels, as well as the mechanisms of nucleocytoplasmic transport. Improvement in imaging and crystallography methods have resulted in significant advances in the ultrastructural characterization of NPCs.^9,16^ Similarly, studies that identified transport receptors, characterized their interactions with nuclear pore complex components, and elucidated physical properties of the NPC transport channel, have resulted in the proposal of nuclear transport models that explain how molecules are shuttled between the nucleus and the cytoplasm.^17–19^ But perhaps one of the most interesting aspects of these structures that has been uncovered in the last decade is the identification of transport-independent functions of NPCs and its components. Among these, the NPC functions that have been studied in more detail are the regulation of genome organization and gene expression.^4^

Since its first description, the role of NPCs in transcriptional regulation has continuously bounce between the positive and negative modulation of gene expression. While early studies in yeast showed that NPCs were associated with silent telomeric and subtelomeric chromatin,^20,21^ therefore suggesting a role of NPCs in gene silencing, more recent studies have shown that many nucleoporins associate with the promoter of active genes, and that NPCs can act as transcriptional activators.^22,23^ In yeast, several genes have been shown to move from the nuclear interior to NPCs in response to activation, and it has become clear that NPC-tethering is important for proper gene expression and transcriptional memory.^4,23–25^ The association of several of these genes with NPCs is mediated by DNA sequences called Gene Recruitment Sequences (GRSs) that are present in their promoters.^26^ In some cases, these sequences represent binding sites for transcription factors, several of which have been shown to play a pivotal role in the association with nuclear pores.^27^ Notably, genes that have the same GRSs can cluster at the nuclear periphery, which might suggest a shared transcriptional regulatory mechanism.^28^ Other factors that have been shown to play a central role in the association of genes with NPCs and in their transcriptional modulation are the SAGA, TREX and mediator complexes.^29–33^ These complexes link transcriptionally active genes to NPCs and the transcription core machinery.^29,33^

In metazoans, the nuclear pore and its components have also been linked to the regulation of gene activity. But until recently, most evidence pointed to the regulation of gene expression by nucleoporins inside the nucleus and not at the nuclear periphery.^34,35^ Initial studies of nucleoporin-gene association in flies found that some nucleoporins, such as Nup98, bind and regulate the activity of genes away from NPCs.^34,35^ Although, NPC-associated Nup98 also bound several genes, these were found to be mostly non-active or have basal/low activity.^35^ Moreover, the first NPC-chromatin interaction studies in mammalian cells suggested that NPCs mostly associate with silent chromatin.^36^ Opposing this original view of NPCs having a repressive role in transcriptional regulation in metazoans, recent studies have uncovered that NPCs also associate with specific genes groups and positively or negatively modulate their activity.^11,37–40^ These functions of nuclear pore complexes as both, activators and repressors of gene activity, is consistent with their ability to bind active and silent chromatin and suggest that this giant structure allows the creation of different localized domains with opposing transcriptional functions. But: 1) the fact that NPCs are mostly surrounded by decondensed chromatin,^41,42^ which these structures actively help to maintain;^43,44^ 2) that superenhancer regions associate with them;^40^ and 3) the recent findings that genes at NPCs require specific nucleoporins for proper expression,^11,37,40^ suggest that the role of NPCs in the active regulation of gene expression in metazoans might so far have been underestimated.

## NPCs as specialized scaffolds for gene expression

We recently identified that during myogenesis there is a change in the composition of NPCs.^15^ This change, the addition of nuclear membrane nucleoporin Nup210, is required for myoblast differentiation and survival.^15^ Consistent with an important role in the physiology of skeletal muscle, depletion of Nup210 during Zebrafish development results in a highly abnormal muscle structure with disorganized and missing muscle fibers.^11^ These alterations in skeletal muscle are a consequence of the inability of Nup210-depleted animals to mature their differentiated cells, which results in apoptotic muscle cell death and the deterioration of muscle tissue.^11^ Notably, we discovered that Nup210 addition to nuclear pores does not affect nucleocytoplasmic transport but is required for the proper regulation of muscle genes.^11,15^ But, how does a transmembrane nucleoporin regulates gene activity? Our findings indicate that Nup210 helps to recruit the transcription factor Mef2C to the nuclear periphery where it regulates a subset of its target genes.^11^ Mef2C is a central regulator of skeletal and cardiac muscle.^45^ Similar to Nup210, depletion of Mef2C leads to the deterioration of differentiated muscle fibers.^46,47^ The Nup210/Mef2C complex only assembles during myogenesis, where Nup210 becomes expressed and localizes to NPCs. This complex is required for the proper expression of structural and other muscle genes that play a role in myofiber maturation and survival. Interestingly, several of the genes we have found to be co-regulated by Nup210 and Mef2C at NPCs are present at the nuclear periphery even in myoblast where Nup210 is not expressed and Mef2C is present at very low levels.^11^ This indicates that this change in NPC composition that occurs during myoblast differentiation is not required for gene-tethering to the nuclear periphery but for the recruitment of transcriptional regulators that modulate the activity of target genes that are already present in the vicinity of nuclear pores. Our findings indicate that NPCs act as scaffolds for the organization of transcription hubs that regulate groups of related genes, sarcomeric and structural genes in muscle for example, and that the activity of these genes can be modulated by changing NPC composition. The presence transcriptional hubs at NPCs might explain the association of superenhancers with these structures.^40^ It is possible to envision that the colocalization of genes that share regulatory elements in the confined space around nuclear pores will expose them to a transcription-permissive chromatin environment where specific transcriptional regulators can be enriched by interaction with NPCs. These conditions might allow for the concert modulation of highly related genes. This role of NPCs as scaffolds for the local organization and regulation of genes groups seems to be conserved in Drosophila, where the nucleoporin Nup98 was found to mediate the enhancer-promoter association of several poised genes tethered to NPCs.^48^ Whether the genes that associate with mammalian NPCs are also poised when non-active remains to be determined.

**Figure 1.**
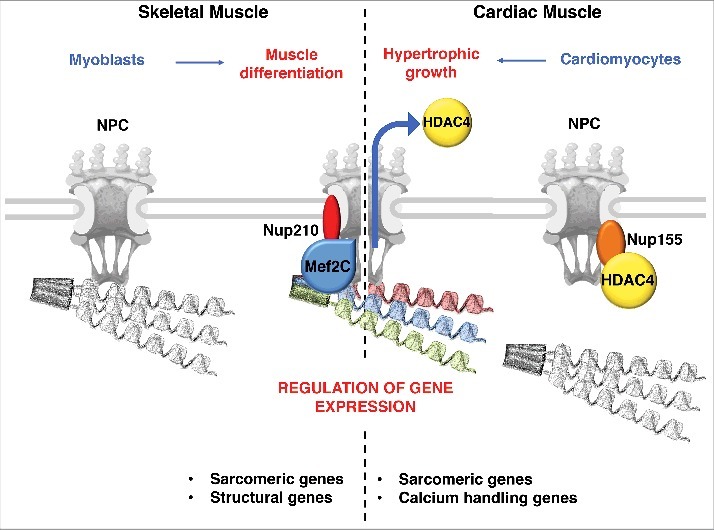
NPCs as hubs for the regulation of muscle genes. Left panel: Schematic illustration of NPC regulation of muscle gene expression in skeletal muscle. During myogenesis, the expression of Nup210 is induced, and this nucleoporin is added to NPCs. In differentiated muscle cells, Nup210 recruits the transcription factor Mef2C to regulate sarcomeric and muscle structural genes that are associated with nuclear pores. Right panel: Schematic illustration of NPC regulation of muscle gene expression in cardiac muscle. In resting cardiomyocytes, the histone deacetylase HDAC4 present at NPCs prevents the association of sarcomeric and calcium-handling genes with these structures. When hypertrophic growth is stimulated, HDAC4 is exported from the nucleus, allowing the association of these genes with NPCs and promoting their efficient transcription.

Mef2C is negatively regulated by the histone deacetylase HDAC4. Notably, in resting cardiomyocytes, HDAC4 was shown to negatively control the association of structural and calcium signaling genes with NPCs.^37^ When hypertrophic growth is induced in these cells, HDAC4 is released from NPCs allowing gene association and activation (Fig. 1). The HDAC-dependent modulation of NPC gene tethering might explain previous findings showing that non-specific inhibition of histone deacetylases using TSA changes the genomic regions that associate with NPCs, favoring active chromatin domains and differentially expressed genes.^36^ Whether HDAC4 plays a role in gene regulation by Nup210/Mef2C and vice versa have not been investigated but it is very likely that muscle gene regulation at NPCs involves a dynamic interplay between these factors. If HDAC4 and Mef2C indeed work together to regulate gene expression at NPCs, the contradicting findings that Nup210 is not required for the tethering of muscle genes to NPCs while HDAC4 it is, could indicate the existence of two separate groups of genes, one stably bound to NPCs and another dynamically associated with the structure; or less likely, differences in the two cell types, skeletal vs cardiac muscle cells.

Another player in the Nup210 regulation of muscle physiology is the LIM-domain protein Trip6,^11^ which shuttles between focal adhesions and the nuclear interior and can either acts as a transcriptional co-activator or co-repressor depending on its associated cofactors.^49^ We found that in post-mitotic muscle cells Trip6 interacts with Nup210 to regulate muscle physiology.^11^ Our findings suggest that a complex of Trip6, Mef2C and Nup210 positively regulates muscle gene expression at NPCs.^11^ Notably, in proliferating myoblasts that do not have Nup210 and have very low levels of Mef2C, Trip6 has been found to interact and act as a co-repressor for Mef2C by mediating the recruitment of HDAC5 to its target genes.^50^ This mechanism might help to further ensure that Mef2C targets are not activated in undifferentiated muscle progenitors. Even though Trip6 represses Mef2C targets in myoblasts,^50^ we have found that like Nup210 and Mef2C this factor is required for myoblast differentiation and can rescue some of the muscle phenotypes of Nup210 depleted animals.^11^ These findings indicate a dual function of Trip6 during myogenesis. Whether Nup210 modulates the repressor/activator functions of Trip6 remains to be investigated.

## Perspective

Over the last two decades, the view of the eukaryotic cell nucleus has changed from that of a simple “container” of the cellular genome, to that of a highly organized cellular organelle with a large number of compartmentalized domains with specialized functions. It has become clear that chromosomes occupy defined spaces within the nucleus, that the 3D organization of the genome influences gene activity, and that certain genes that are co-regulated congregate in specialized domains known as transcription factories. Nuclear pore complexes are the largest protein complexes of the nucleus. Thus, it is not surprising that we are finding more and more evidence that these structures play an important role in organizing and controlling the function of the eukaryotic genome. Particularly interesting is the fact that these macromolecular channels are emerging as organizers of localized transcriptional hubs. So far, the functional relevance of only a few transcriptional regulators that associate with NPCs has been characterized but it is foreseeable that in the next decade many more will be unveiled. This will likely solidify the role of NPCs as scaffolds for the assembly of localized transcription factories and further support the idea that two different gene-regulatory environments exist at the nuclear periphery, an active NPC-associated compartment and a repressive lamina-associated one.
